# Excess CD40L does not rescue anti-DNA B cells from clonal anergy

**DOI:** 10.12688/f1000research.2-218.v2

**Published:** 2014-01-15

**Authors:** Mohammad Aslam, Yusuke Kishi, Takeshi Tsubata

**Affiliations:** 1 Department of Immunology, Medical Research Institute, Tokyo Medical and Dental University, Tokyo, 113-8510, Japan

## Abstract

CD40L, a member of the tumor necrosis factor (TNF) ligand family, is overexpressed in patients with systemic lupus erythematosus and in lupus mouse models. Previously, we demonstrated that B cells producing pathogenic anti-Sm/RNP antibodies are deleted in the splenic marginal zone (MZ), and that MZ deletion of these self-reactive B cells is reversed by excess CD40L, leading to autoantibody production. To address whether excess CD40L also perturbs clonal anergy, another self-tolerance mechanism of B cells whereby B cells are functionally inactivated and excluded from follicles in the peripheral lymphoid tissue, we crossed CD40L-transgenic mice with the anti-DNA H chain transgenic mouse line 3H9, in which Ig λ1+ anti-DNA B cells are anergized. However, the percentage and localization of Ig λ1+ B cells in CD40L/3H9 double transgenic mice were no different from those in 3H9 mice. This result indicates that excess CD40L does not perturb clonal anergy, including follicular exclusion. Thus, MZ deletion is distinct from clonal anergy, and is more liable to tolerance break.

## Introduction

Antibodies to nuclear antigens such as DNA and the RNA-related Sm/RNP antigen are characteristically produced in patients with systemic lupus erythematosus (SLE), a prototype of systemic autoimmune diseases, and play a role in the development of this autoimmune disease ^1– 3^. How B cells reactive to nuclear antigens are regulated has been extensively studied using transgenic (Tg) mice expressing auto-antibodies against DNA and RNA components, especially the anti-DNA H chain-Tg mouse lines 3H9 and 56R ^4– 9^. Studies using these auto-antibody-Tg mice demonstrated that self-reactive B cells that produce autoantibodies to nuclear antigens are deleted by apoptosis (clonal deletion) ^10^, are functionally inactivated (clonal anergy) ^11^ or change antigen specificity by immunoglobulin (Ig) V gene replacement (receptor editing) ^8, 12^, in the bone marrow before they migrate to the peripheral lymphoid organs. These self-tolerance mechanisms appear to be involved in the prevention of autoantibody production in normal individuals.

CD40 is a member of the tumor necrosis factor (TNF) receptor family expressed in immune cells including B cells and dendritic cells ^13^. Upon interaction with its ligand, CD40L (CD154), expressed mainly by activated T cells, CD40 transmits survival and activation signals in B cells ^13, 14^. In both patients with SLE and in SLE mouse models, CD40L is overexpressed by T cells and ectopically expressed in B cells ^15– 17^, and this excess CD40L expression appears to play a role in development of SLE, as treatment with antagonistic anti-CD40L antibody markedly reduces the severity of the disease in both humans and mice ^18^.

Using CD40L/56R double transgenic mice expressing both the anti-DNA H chain 56R and CD40L in B cells, we previously demonstrated that anti-Sm/RNP B cells are regulated by a novel tolerance mechanism in peripheral lymphoid tissue, i.e., deletion in splenic marginal zone (MZ), and that the MZ deletion is perturbed by excess CD40L ^19^. In 56R mice, B cells that produce anti-Sm/RNP antibody appear in the splenic MZ, and are subsequently deleted there by apoptosis. When 56R mice are crossed with CD40L-Tg mice in which CD40 signaling is constitutively generated in B cells ^20^, MZ deletion of anti-Sm/RNP B cells is perturbed, resulting in autoantibody production ^19^. As anti-Sm/RNP antibodies are implicated in the pathogenesis of SLE ^1, 2^, MZ deletion appears to be important for preventing the development of SLE through deletion of pathogenic self-reactive B cells. Hence, a defect in MZ deletion by excess CD40L ^19^ could play a role in the development of lupus by inducing the production of pathogenic anti-Sm/RNP antibody.

Some self-reactive B cells including a part of anti-DNA B cells are silenced by clonal anergy in which B cells persist in the peripheral lymphoid organs but are unresponsive to antigen stimulation. Anergized self-reactive B cells are excluded from follicles or the MZ of the spleen. Instead, they are located in the red pulp and the T cell zone of the spleen, especially in the border between the T cell zone and the follicles, and undergo apoptosis ^21^. Ig λ1 L chain ^+^ B cells in the anti-DNA H chain-Tg mouse line 3H9 are reactive to DNA and are anergized ^9, 22, 23^. Previously, we demonstrated that excess CD40L does not induce anti-DNA antibody production in 3H9 mice ^19^. Nonetheless, it is possible that anti-DNA B cells in these mice are rescued from anergy by excess CD40L but are tolerized by some other mechanism as there are multiple tolerance mechanisms at different developmental stages of B cells. To address whether excess CD40L can reverse the anergy of self-reactive B cells, we crossed CD40L-Tg mice with 3H9 mice and examined the percentage and localization of Ig λ1 ^+^ B cells. Our results demonstrated that excess CD40L does not expand anergized anti-DNA B cells or reverse their follicular exclusion, indicating that excess CD40 does not reverse anergy of self-reactive B cells. As excess CD40L does perturb MZ deletion of anti-Sm/RNP B cells, clonal anergy appears to be distinct from MZ deletion, although both of them induce apoptosis of self-reactive B cells in peripheral lymphoid tissue.

## Materials and methods

### Mice

The conventional Tg mouse line expressing the H chain of the anti-DNA antibody 3H9 on the BALB/c background ^5^ was a kind gift of Dr. M. Weigert (The University of Chicago). We previously generated CD40L-Tg mice on the C57BL/6 background ^20^. CD40L-Tg mice were crossed with 3H9-Tg mice to generate wild type, 3H9-Tg and CD40L/3H9 double Tg mice on (BALB/c × C57BL/6)F1 background. Mice were genotyped by PCR reaction using specific pairs of primers for the CD40L and 3H9 transgenes, respectively ^14, 19^. All mice were housed and bred at our specific pathogen-free facility. Groups of 3 mice were kept in conventional shoebox-type polycarbonate cages, which were changed every 7 days. All animals were provided with food (CE-2, CLEA Japan, Inc.) and water *ad libitum* and were maintained on a 12-hour light/dark cycle. All procedures followed the guidelines of Tokyo Medical and Dental University for animal research and were approved by Institutional Animal Care and Use Committee, Tokyo Medical and Dental University. For each of 3H9, CD40L and CD40L/3H9-Tg mice, 3 or 4 female mice at 11–34 weeks of age were analyzed by flow cytometry and immunohistochemistry, respectively. Mice were euthanized by cervical dislocation after CO2-induced unconsciousness and whole spleens were removed aseptically.

### Flow cytometry

Spleens were finely minced over a wire mesh, and spleen cells were collected and suspended in PBS containing 2% FCS (Cell Culture Bioscience, Japan) and 0.1% NaN _3_ ( Nacalai Tesque, Inc., Japan). Cells (1 × 10 ^7^/ml) were then stained with the following antibodies and reagents. The dilutions at which the antibodies were used are indicated in parentheses. Alexa Fluor 647-conjugated rat anti-mouse B220 (RA3-6B2, BioLegend, USA) (1:100), Pacific Blue-conjugated anti-mouse Ig λ1 (LS-136, a kind gift of Dr. G. Kelsoe at Duke University) (1:100) ^24^ and FITC-conjugated goat anti-mouse Ig λ chain antibody (Cat No. 1060-02, Southern Biotech, USA) (1:1000). Lymphoid cells were gated by FSC/SSC dot plots, and analyzed on a CyAn ADP flow cytometer (Beckman Coulter, USA).

### Immunohistochemistry

Tissues were embedded in Tissue-Tek O.C.T. compound (Sakura Finetek U.S.A., Inc., U.S.A.), snap-frozen in liquid nitrogen, and stored at −80°C. Cryostat (Cryostat 1720, Leica Microsystems GmbH, Germany) sections of 5 μm in thickness were mounted onto micro slide glass (Matsunami Glass Ind. Ltd., Japan), air dried, and fixed in acetone at room temperature for 20 min. The sections were incubated with blocking buffer (PBS containing 2.0% (wt/vol) FCS (Cell Culture Bioscience, Japan), 0.05% NaN3 ( Nacalai Tesque, Inc., Japan)) for 30 min and were stained at room temperature for 60 min with biotin-conjugated rat anti-mouse MOMA-1 (Cat No. T-2021, BMA Biomedicals, Switzerland), Alexa Fluor 647-conjugated streptavidin (Cat No. S21374, Invitrogen, USA), Pacific Blue-conjugated rat anti-mouse B220 (RA3-6B2, BioLegend, USA) and FITC-conjugated goat anti-mouse λ chain antibody (Cat No. 1060-02, Southern Biotech, USA). All the antibodies and reagents were used at a 1:100 dilution.

### Statistical analysis

Spleen cells were compared between animals. Statistical analysis of data by two-tailed Student *t* test was performed using Prism 5.0 software (GraphPad). *p* < 0.05 was considered statistically significant.

## Results

### Excess CD40L fails to expand anergized anti-DNA B cells

**Figure 1.**
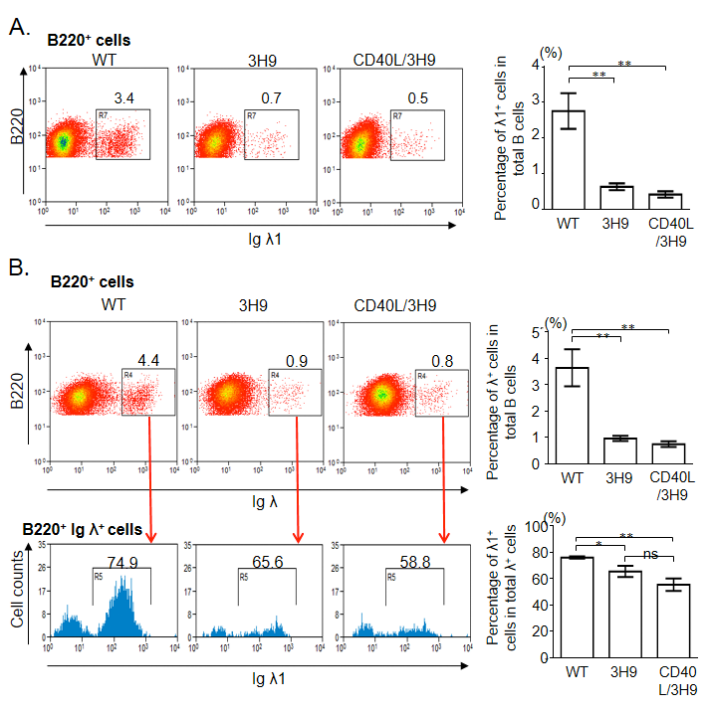
Excess CD40L does not expand anergic anti-DNA B cells in 3H9 mice. Spleen cells from 11- to 34-wk-old wild type (WT), 3H9, and CD40L/3H9 mice were stained for B220 ( **A** and **B**), Ig λ1 chain ( **A** and **B**) and Ig λ chain ( **B**), and B220 ^+^ cells were analyzed by flow cytometry. Percentages of λ1 ^+^ cells in total B220 ^+^ cells ( **A**), percentages of λ ^+^ cells in total B220 ^+^ cells ( **B**, upper panel) and percentages of λ1 ^+^ cells in total λ ^+^ cells ( **B**, lower panel) are indicated (left panels). Representative data from three independent experiments. Graphs show mean ± SD, three mice per genotype (right panels). * p<0.05, ** p<0.01, ns = not significant.

To address whether excess CD40L perturbs clonal anergy of anti-DNA B cells, we crossed CD40L Tg mice with 3H9 mice, because B cells expressing Ig λ1 L chain and 3H9 H chain are reactive to DNA and show characteristics for anergy including follicular exclusion, shortened life span and failure of antibody production ^23^. When spleen B cells of wild type, 3H9 Tg and CD40L overexpressing CD40L/3H9 double Tg, mouse lines were analyzed by flow cytometry, the percentage of λ1 ^+^ B cells in total B cells expressing a B cell marker B220 was markedly reduced in 3H9 mice compared to wild type mice ( Figure 1A) (p < 0.01) as λ1 ^+^ B cells in 3H9 but not wild type mice are self-reactive. The percentage of λ1 ^+^ B cells in CD40L/3H9 double Tg mice was equivalent to that in 3H9 mice, indicating that excess CD40L does not expand anergized anti-DNA B cells.

When we used anti-λ antibody that reacts to multiple Ig λ chain subtypes such as λ1 and λ2, the percentage of total λ ^+^ cells were only slightly higher than that of λ1 ^+^ cells in wild type, 3H9 and CD40L/3H9 mice ( Figure 1A and B upper panels), suggesting that most of the λ ^+^ cells express the λ1 subtype in all these mice and are thus reactive to DNA. This is confirmed by determining percentage of λ1 ^+^ cells in total λ ^+^ cells. In both 3H9 and CD40L/3H9 mice, the percentage of λ1 ^+^ cells in total λ ^+^ cells is slightly reduced compared to that in wild type mice (p < 0.05 and 0.01, respectively), but λ1 ^+^ cells constitute the majority of the λ ^+^ cells in these mice as well as wild type mice ( Figure 1B lower panels).

### Excess CD40L does not reverse follicular exclusion of anergized anti-DNA B cells

**Figure 2.**
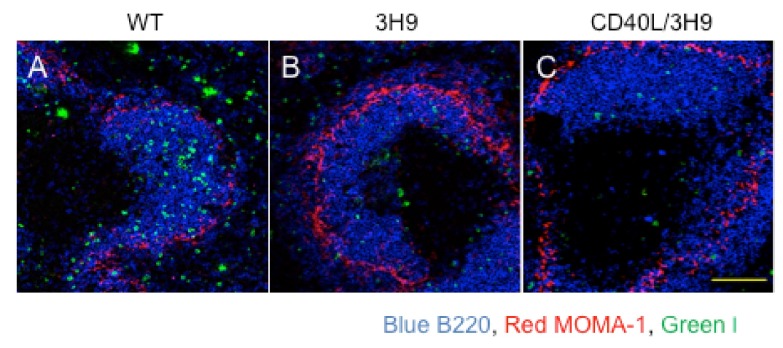
Excess CD40L does not reverse follicular exclusion of anergic anti-DNA B cells in 3H9 mice. Sections of spleen from 11- to 34-wk-old wild type (WT) ( **A**), 3H9 ( **B**), and CD40L/3H9 ( **C**) mice were stained for MOMA-1, a marker for a subset of splenic macrophages (red), B220, a B cell marker (blue), Ig λ L chain (green), and analyzed by confocal microscopy. Ig λ ^+^ B cells in 3H9 mice are DNA-reactive. B cells positive for B220 are located in the follicles, and MOMA-1 ^+^ macrophages are located at the outer part of the follicles. T cell zone and red pulp are located inside and outside of the follicles, respectively. Representative data from four independent experiments. Four mice in each group were analyzed. Original magnifications, 20X. Scale bar (yellow line): 100 μm.

To address whether excess CD40L reverses follicular exclusion of anergized anti-DNA B cells, we examined spleen sections of 3H9 and 3H9/CD40L Tg mice using an anti-λ antibody but not anti-λ1 antibody, as the anti-λ1 antibody did not yield any staining when used for immunohistochemistry. Nonetheless, most of the B cells stained with anti-λ antibody in these mice express λ1 ( Figure 1B, lower panels) and are thus reactive to DNA. In wild type spleen, λ ^+^ cells were located mostly in the follicle where B220 ^+^ B cells accumulate ( Figure 2A). In contrast, λ ^+^ cells were found mostly in the border between T cell zone and follicle, T cell zone and red pulp in both 3H9 and CD40L/3H9 spleens ( Figure 2B and C), indicating that anti-DNA B cells are excluded from follicles in CD40L/3H9 mice as well as 3H9 mice. Thus, excess CD40L does not reverse follicular exclusion of anergized anti-DNA B cells.

Flow cytometry data of spleen cells from wild type, 3H9-transgenic and CD40L/3H9 double transgenic mice Data 1. Flow cytometry analysis of spleen cells from wild type, 3H9-transgenic and CD40L/3H9 double transgenic mice. Spleen cells were obtained from wild type (A and D), 3H9-transgenic (B and E), and CD40L/3H9 double transgenic mice (C and F). Cells were stained with Alexa Fluor 647-conjugated (FL 8 channel) rat anti-mouse B220 and Pacific Blue-conjugated (FL 6 channel) anti-mouse Ig λ1(LS-136), and with (D-F) or without (A-C) FITC-conjugated (FL 1 channel) goat anti-mouse Ig λ chain. Lymphoid cells were gated by FSC/SSC dot plots and the B220+ cells were analyzed on a CyAn ADP flow cytometer (Beckman Coulter, USA).

Microscopy data of spleen cells from wild type, 3H9-transgenic and CD40L/3H9 double transgenic mice Data 2. Fluorescent microscopic analysis of spleen sections from wild type, 3H9-transgenic and CD40L/3H9 double transgenic mice. Spleen sections were obtained from wild type (A), 3H9-transgenic (B), and CD40L/3H9 double transgenic mice (C). Tissue sections were stained with biotin-conjugated rat anti-mouse MOMA-1, Alexa Fluor 647- conjugated streptavidin (red), Pacific Blue-conjugated rat anti-mouse B220 (green) and FITC-conjugated goat anti-mouse λ chain (blue) antibody. Fluorescent images were obtained with a Zeiss LSM 510 META laser scanning confocal microscope.

## Discussion

In this study, we crossed CD40L-Tg mice with anti-DNA H chain-Tg 3H9 mice, and demonstrated that the percentage and location of λ1 ^+^ anergic anti-DNA B cells are not altered in CD40L/3H9 double Tg mice compared to those in 3H9 mice, suggesting that anergy of λ1 ^+^ anti-DNA B cells is not reversed in CD40L/3H9 mice. This result is consistent with our previous finding that CD40L/3H9 mice do not produce anti-DNA antibodies in sera, whereas another anti-DNA H chain Tg mouse line 56R in which self-reactive B cells are deleted at MZ did produce autoantibodies in sera when crossed with the same CD40L Tg mice ^19^. Thus, excess CD40L does not perturb anergy of anti-DNA B cells.

Although excess CD40L fails to perturb clonal anergy of anti-DNA B cells in 3H9 mice, anergic B cells are not insensitive to CD40L. A previous study by Lesley *et al.* demonstrated that anergic self-reactive B cells expand by receiving stimulation from a low level expression of CD40L in unstimulated T cells ^25^. Thus, anergic B cells sensitively respond to a low level CD40L expression, but do not further respond to excess CD40L expression. The mechanisms behind this phenomenon however, are not yet clear.

Using the same CD40L-Tg mice that we used in the present study, we previously demonstrated that excess CD40L inhibits apoptosis of anti-Sm/RNP B cells in MZ, and that excess CD40L induces autoantibody production ^19^. This suggests that MZ deletion is distinct from clonal anergy, although both anergic B cells and B cells that are deleted in MZ appear in peripheral lymphoid tissue and are eliminated by apoptosis.

Like CD40L, B cell activating factor (BAFF), another member of the TNF ligand family, induces B cell survival and is overexpressed in patients with SLE ^26– 28^ and its mouse models ^29, 30^. Anergic B cells show increased dependency on BAFF for survival, and this increased dependency appears to be involved in rapid elimination of anergic B cells by competition with non-self-reactive B cells ^31, 32^. In the presence of non-self-reactive B cells, anergic B cells may not be able to interact with a sufficient level of BAFF required for their survival, due to competition for a limited amount of BAFF ^32^. Nonetheless, excess BAFF fails to fully reverse anergy of self-reactive B cells. Lesley *et al*. ^31^ demonstrated that excess BAFF expands anergic B cells but fails to reverse follicular exclusion. Thien *et al*. ^32^ demonstrated that excess BAFF expands and reverses follicular exclusion in only anergic B cells with intermediate affinity but not those with high affinity. In contrast, cognate T cell help perturbs follicular exclusion and induces autoantibody production in anergic self-reactive B cells ^33^. Thus, reversing clonal anergy requires strong T cell help, and excess BAFF or CD40L alone may be insufficient. In contrast, we previously demonstrated by crossing the CD40L-Tg mice with another anti-DNA H chain Tg mice 56R that excess CD40L perturbs MZ deletion of self-reactive B cells and induces autoantibody production, suggesting that MZ deletion is more sensitive to a tolerance break than clonal anergy ^19^. As excess CD40L is found in patients with SLE and various SLE model mice, MZ deletion is likely to be defective in lupus, and its defect may be involved in development of lupus.
