# Supplementary material for: Excess CD40L does not rescue anti-DNA B cells from clonal anergy
Source: F1000Res. 2014 Jan 15;2:218. Originally published 2013 Oct 17. [Version 2] doi: 10.12688/f1000research.2-218.v2 (PMC3907155; doi:10.12688/f1000research.2-218.v2)
Supplement: Peer review history

# Peer review: Referee response for version 2

**DOI:** 10.5256/f1000research.3592.r3145

**Published:** 2014-03-05

**Authors:** Jennifer Marshall

**Affiliations:** 1 University of Birmingham, Birmingham, UK

With the addition of the animal numbers used, the graphs of data in figure 1 and the new images submitted I think that the data, and therefore the paper, is much improved. I would very much like to thank the authors for making these changes.

I have read this submission. I believe that I have an appropriate level of expertise to confirm that it is of an acceptable scientific standard.

# Peer review: Referee response for version 2

**DOI:** 10.5256/f1000research.3592.r3359

**Published:** 2014-01-28

**Authors:** David Nemazee

**Affiliations:** 1 Department of Immunology and Microbial Science, Scripps Research Institute, La Jolla, CA, USA

The paper has been much improved, as it now provides more data on the statistical significance of the findings. Overall, this is a straightforward, though somewhat limited study. I now find it acceptable without further changes.

I have read this submission. I believe that I have an appropriate level of expertise to confirm that it is of an acceptable scientific standard.

# Peer review: Referee response for version 1

**DOI:** 10.5256/f1000research.2308.r2590

**Published:** 2013-11-28

**Authors:** David Nemazee

**Affiliations:** 1 Department of Immunology and Microbial Science, Scripps Research Institute, La Jolla, CA, USA

The paper by Aslam, Kishi and Tsubata addresses an interesting question about the effects of bystander stimulation through CD40L on immunological self tolerance of B cells. Previous studies from this group showed that transgene-enforced CD40L expression on B cells promoted a lupus-like autoimmunity in mice with a polyclonal B cell population. A later study showed that autoreactive B cells in a transgenic mouse with a restricted antibody repertoire biased to DNA reactivity (mice carrying 3H9-56R DNA-reactive H-chain) were promoted to central tolerance in the presence of excess CD40L, whereas 3H9-H mice lacking that mutation, while still expressing a repertoire biased to autoreactivity, failed to show a clear break in tolerance.

The present study is a rather minor addition to this body of work, providing additional data on the fate of B cells expressing a λ1 light chain partner, which have high anti-DNA affinity when expressed with 3H9H. The data show that 3H9H/λ1 B cells are not rescued in their development or altered in their anatomical localization (follicular exclusion) when developing in an environment with excess B cell-expressed CD40L. The data are consistent with the earlier, more extensive study, but do provide some new information. However, the data shown have no indication of reproducibility or provide more than a single mouse per group. There is no molecular characterization of the cells to verify that the receptor expression expected is indeed confirmed. Overall, this is an incremental contribution to the literature.

I have read this submission. I believe that I have an appropriate level of expertise to state that I do not consider it to be of an acceptable scientific standard, for reasons outlined above.

# Peer review: Referee response for version 1

**DOI:** 10.5256/f1000research.2308.r2424

**Published:** 2013-11-25

**Authors:** Jennifer Marshall

**Affiliations:** 1 University of Birmingham, Birmingham, UK

This report from Aslam *et al.*, provides data on the lack of an effect of over-expression of CD40L on the number and location of anergic 3H9 B cells that respond to DNA. This follows on from their 2012 PNAS study which used similar methods investigating the effects of CD40L on anergic B cells from 56R mice.

The paper is well constructed and all sections are clear. However, I believe that the data could be presented in a manner more helpful to the reader and provide a firmer foundation for their conclusions. I think that these changes (especially those concerning figure 2) are necessary to substantiate their conclusions and abstract.

Both figure legends should contain information on how many mice were used in each group. This information is essential for all publications involving animal work and is necessary to allow proper judgment of data. Whilst the methods do state that the mice are caged in groups of 3 this is within the details of animal husbandry and, as such, it is not clear if this related to the experimental group size.

In addition to the representative flow cytometry plots provided Figure 1A and B would benefit from a graph that shows the percentage of splenic Ig λ1 L chain B cells of each mouse in the experiment. This would provide an opportunity to be made aware of the variation between the mice within each group as well as compare between groups. If there are sufficient mouse numbers a statistical analysis comparing groups would also be useful.

I am puzzled by the choice of images for Figure 2. Firstly, due to the shape of the follicle it is difficult to compare the location of Ig λ L chain cells in 2C with those in 2A and B. As the follicular exclusion of λ L chain B cells within the CD40L/3H9 mouse is a key conclusion of this paper it would be helpful for the reader if 2C was replaced with a representative white pulp area that provides a clearer demarcation between follicle and the T zone. Secondly, I would suggest that Figure 2B makes it appear that the 3H9 mice have substantially fewer Ig λ L chain B cells than the CD40L/3H9 mice shown in Figure 2C. If it is representative of all 3H9 spleens I do not think that this corroborates the conclusions drawn from Figure 1. As they stand the images make my suggestions regarding an extra graph for Figure 1, and the mouse numbers used, more compelling.

Without these changes I cannot truly say that the title and conclusions are appropriate. However, these are quick changes and I trust that it is just a bad image choice.

I have read this submission. I believe that I have an appropriate level of expertise to confirm that it is of an acceptable scientific standard, however I have significant reservations, as outlined above.

# Peer review: Referee response for version 1

**DOI:** 10.5256/f1000research.2308.r2273

**Published:** 2013-10-31

**Authors:** Marko Radic

**Affiliations:** 1 Microbiology, Immunology and Biochemistry, University of Tennessee Health Science Center, Memphis, TN, USA

The brief report by Aslam *et al*. develops a theme that was the initial focus of the Tsubata lab in the early 90’s. The authors follow up on the observation that CD40L is overexpressed on T cells in SLE patients and, ectopically, on B cells. As CD40L provides an important second signal to B cells, the question was whether it can overcome negative regulation of certain B cell states. The current work continues upon Kishi *et al*. (PNAS 2012) who used anti-DNA transgenic mice to examine the effect of CD40L overexpression on the regulation of autoreactive B cells. Kishi *et al.* established that an avid anti-DNA transgene, 56R, led to B cell sequestration in the splenic marginal zone (MZ) and death by apoptosis. CD40L overexpression was able to inhibit apoptosis in the MZ and allow expression of certain H/L pairs, most notably those with anti-Sm specificity. Here, the authors use a closely related anti-DNA H chain, 3H9, which is of lower affinity for DNA compared to 56R.

Dozens of labs have used the 3H9 and the 56R H-chain transgenic mouse lines which have become emblematic for the type of H chain that is immunodominant and – with different L chains – has the potential to encode a range of self-specificities, including anti-DNA, anti-phospholipid, anti-nucleosome and also anti-apoptotic cell antibodies. Earlier work by the Erikson lab had established that 3H9VH in combination with λ1 gives a B cell receptor that induces anergy and leads to a characteristic extrafollicular localization. Aslam *et al*. observe that the 3H9/λ1 combination produces the same number of splenic B cells whether or not it is crossed to CD40L and that the splenic location of the single and double transgenic B cells is identical.

The significance of this work is that it draws an important distinction between tolerance mechanisms that result in anergy vs. MZ deletion. Only the latter is sensitive to rescue by CD40L signals. This serves to clarify important characteristics of splenic B cell regulation. It will be informative to follow these studies with additional approaches to test whether B cell signalling pathways are perturbed by the presence of CD40L, whether increased CD40L expression on B or T cells is involved in suppressing apoptosis in the MZ, and whether CD40L overexpression could affect other types of 56R “incomplete editing” such as in the case of intracellular Golgi retention that is observed with 56R and Vk38c and λX L chains.

The title is appropriate for the content of the article and the abstract represents a suitable summary of the work. 

The design, methods and analysis of the results from the study have been explained and they are appropriate for the topic being studied. The conclusions are sensible, balanced and justified on the basis of the results of the study and enough information has been provided to be able to replicate the experiment.

I have read this submission. I believe that I have an appropriate level of expertise to confirm that it is of an acceptable scientific standard.
